# Secondary Cardiac Involvement From Diffuse Large B-cell Lymphoma: A Case Report

**DOI:** 10.7759/cureus.27664

**Published:** 2022-08-04

**Authors:** Gloria Akimana, Bouthayna Mesmoudi, Mohammed Benasser, Hassan Errihani, Latifa Oukerraj

**Affiliations:** 1 Department of Medical Oncology, National Institute of Oncology, Mohammed V University, Rabat, MAR; 2 Department of Cardiology B, Mohammed V University, Ibn Sina University Hospital Center, Rabat, MAR; 3 Department of Oncology, National Institute of Oncology, Mohammed V University, Rabat, MAR

**Keywords:** involvement, lymphoma, b-cells, diffuse, cardiac, secondary

## Abstract

The cardiac localization of metastases is a rare condition, most often found on autopsy. Lymphoma is among the primary cancers often involved, as was the case for our patient in whom cardiac involvements were discovered during the extension assessment by imaging. Clinically asymptomatic in the majority of cases, we mainly find general symptoms related to the primary disease. Because histological diagnosis is rarely possible by biopsy, confirmation is made by imaging in the context of disseminated disease. The treatment of cardiac involvements of lymphomatous origin is often medical with a poor prognosis.

## Introduction

Although secondary cardiac involvements are rare, they occur more often than primary benign or malignant heart tumors. They occur 20 to 40 times more frequently than primary heart tumors [1]. Rarely described during the lifetime of patients, they have been described in 10.7% of cases in previous autopsy series of cancer patients [2]. Lymphomas are among the most common primary cancers (11.9% for male subjects and 17% for female subjects) [3] as the origin of these involvements. Here, we report the case of a patient whose cardiac involvements were discovered incidentally during an extension workup for non-Hodgkin lymphoma (NHL) by a computed tomography (CT) scan.

## Case presentation

**Figure 1:**
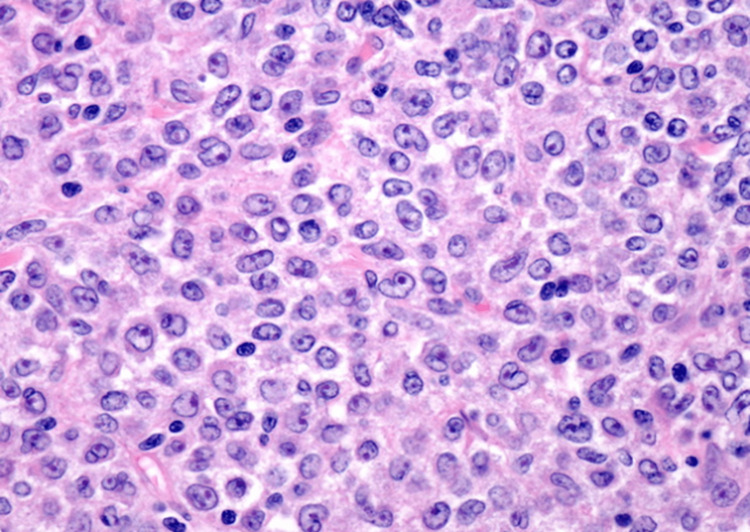
Histological examination: hematoxylin and eosin stain showing large cells with irregular nuclear contours, vesicular chromatin, prominent nucleoli, and moderate cytoplasm.

**Figure 2:**
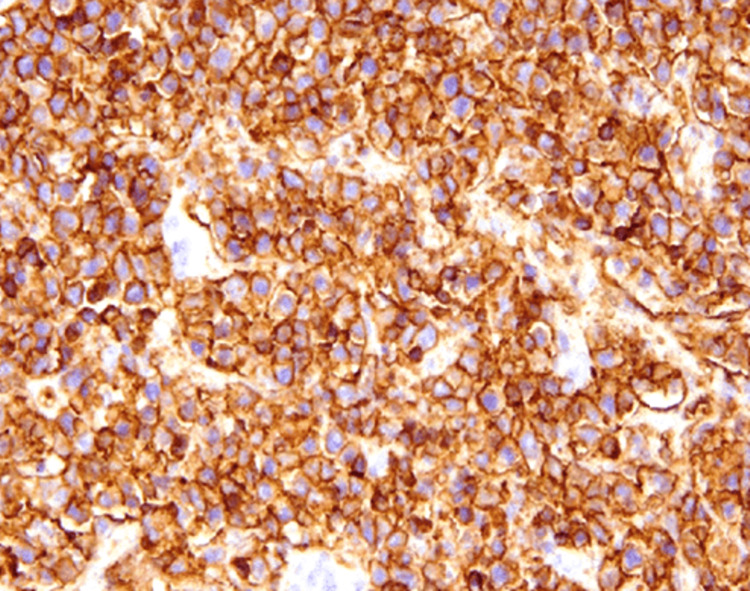
Immunohistochemistry: large cells showing positive staining for CD20.

We report the case of a 49-year-old male patient who underwent emergency surgery for acute bowel obstruction with hail resection. Clinically, the patient presented with non-quantified weight loss, profuse sweating, right inguinal adenopathy of about 3 cm, and sinus tachycardia. Biologically, there was an inflammatory syndrome as well as very high lactate dehydrogenase (817 U/L, almost four times the normal value). The pathological examination showed large cells with irregular nuclear contours, vesicular chromatin, prominent nucleoli, and moderate cytoplasm (Figure 1). On immunohistochemistry examination, the large cells showed positive staining for CD20 (Figure 2), confirming diffuse large B-cell lymphoma (DLBCL). One month later, he presented with a testicular mass along with an inflammatory syndrome. He underwent a right orchiectomy, and histology and immunohistopathology revealed DLBCL.

**Figure 3:**
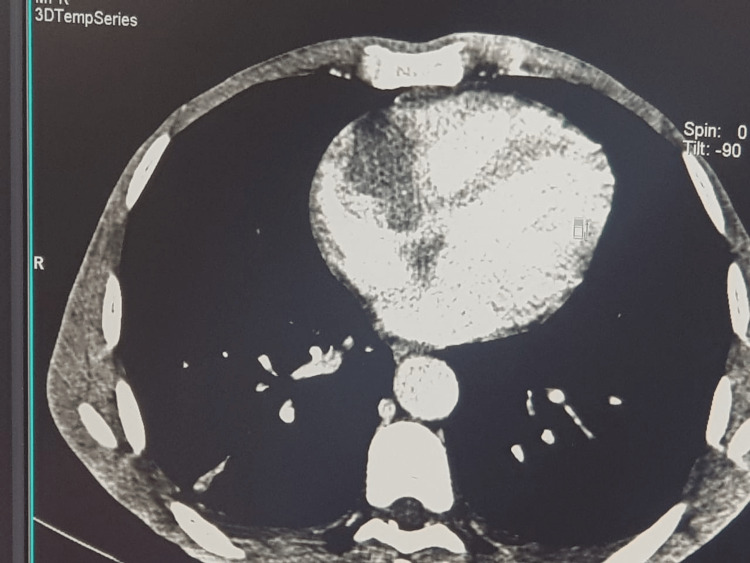
Thoracic computed tomography image showing an endoluminal tissue defect in the right atrium measuring 42 × 38 mm.

An extension assessment by a thoraco-abdomino-pelvic CT scan revealed a 31 mm thick pericardial effusion with individualization of an endoluminal tissue defect in the right atrium measuring 42 × 38 mm, as well as supra- and sub-diaphragmatic adenopathies and hepatic lesions, corresponding to intracardiac and hepatic localizations (Figure 3).

**Figure 4:**
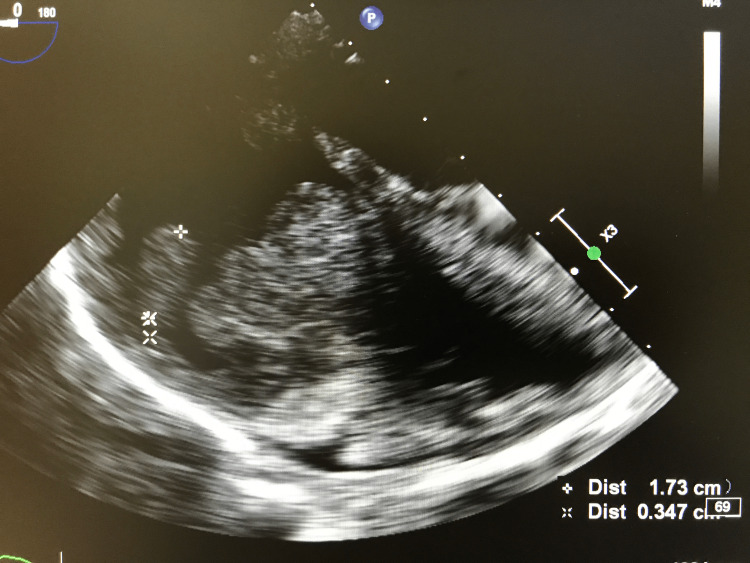
Transesophageal image showing a pedicled mass of the sidewall of the right atrium measuring 17 mm × 7 mm.

Echocardiography revealed a dilated right atrium with two homogeneous masses, one large bilobed pedicled at the interatrial septum measuring 32 × 38 × 41 mm, another one also attached to the interatrial septum measuring 18 × 12 mm, and a third mass of the same texture at the pulmonary artery measuring 7 × 7 mm, along with pericardial effusion of great abundance without respiratory variations. The left ventricular ejection fraction (LVEF) was measured at 65%. The echocardiography was supplemented by a transesophageal ultrasound which revealed a dilated right atrium, seat of multiple regularly contoured masses, the largest of which was oval polylobed with regular contours and heterogeneous content with a wide implantation base at the right atrial sidewall that did not infiltrate and did not interfere with tricuspid valve clearance, measuring 42 mm × 35 mm × 28 mm and infiltrating the entire right atrial sidewall and filling the right auricle. A second ovoid mobile mass with regular contours and homogeneous pedicled content was noted measuring 17 mm × 7 mm with a narrow 3 mm pedicle implanted on the sidewall of the right atrium (Figure 4).

A third round mass with the same structure as the other homogenous and regularly contoured masses with a wide implantation base was implanted at the level of the superior vena cava in the right atrium, measuring 15 mm × 8 mm.

**Figure 5:**
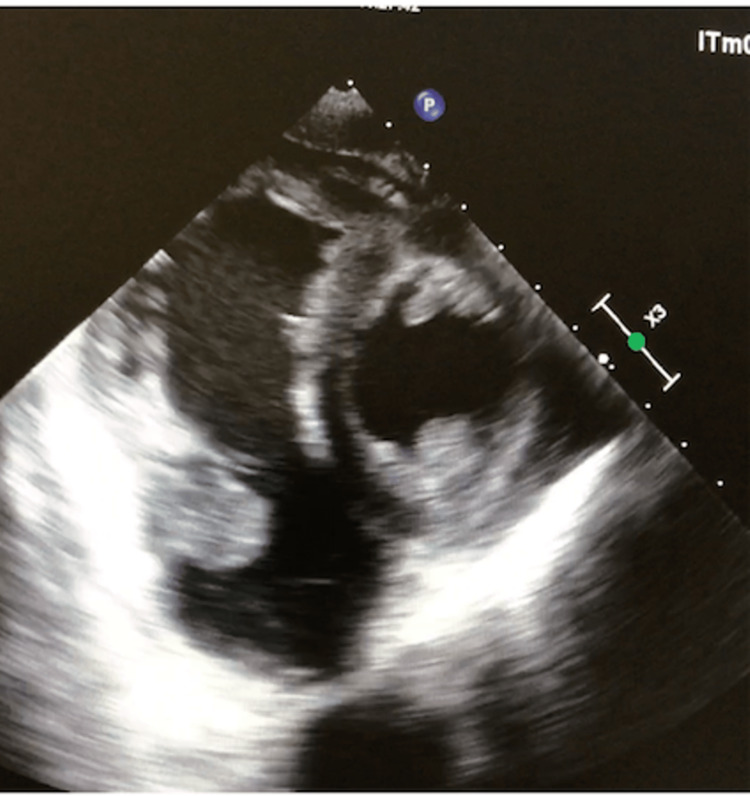
Transesophageal image showing a mass of the right ventricle measuring 14 mm × 13 mm × 12 mm.

**Figure 6:**
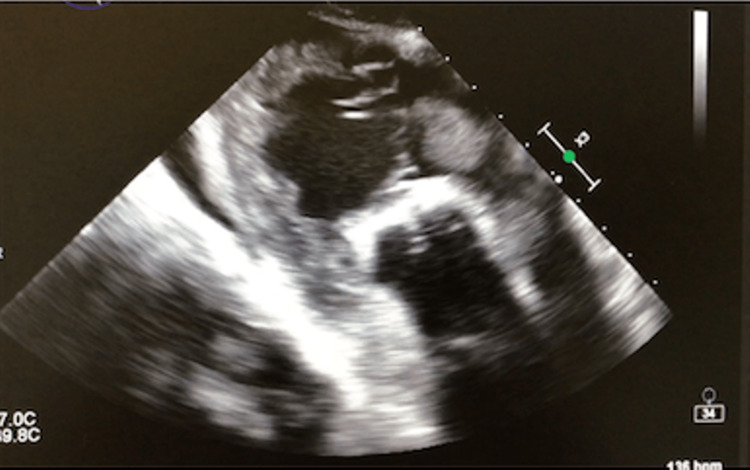
Transesophageal image showing masses on the pulmonary artery ejection pathway.

The right ventricle was hypertrophied with a round, homogenous, regularly contoured mass measuring 14 mm × 13 mm × 12 mm at the level of its flushing chamber, and the pulmonary artery was the site of a small hyperechogenic mass measuring 8 mm × 6 mm before the bifurcation (Figures 5, 6). The tricuspid regurgitation was moderate.

Because of the altered general condition of the patient with a performance status of 2-3, the multiple localization of the disease, and the sensitive nature of the lymphomas, chemotherapy was started urgently with the R-CHOP regimen. Unfortunately, the patient died after the first cure before any imaging evaluation.

## Discussion

Cardiac metastases of cancers are rare, of the order of 10% in autopsy series [2]. The primary tumors most often involved include lung cancer, breast cancer, lymphomas, and melanoma [2,3]. Adenocarcinoma is the most common histological type, while other types are less common [2].

The right heart is most often affected, with involvement of the pericardium first, followed by the myocardium, and rarely the endocardium. This invasion occurs by lymphatic or hematogenous extension, directly by contiguity, or by venous diffusion [1,4,5], and is part of multiple diseases, with the heart being the only metastatic site in only 50% of cases [6].

The clinical presentation of cardiac metastases is often asymptomatic, which differentiates them from primary cardiac involvement where cardiac signs are at the forefront. When symptomatic, the signs that may appear are mainly tachycardia, dyspnea, heart failure, and tamponade. Most often patients only show general and functional signs related to the primary disease [7]. A study of a series of four cases of cardiac localization of NHL found non-specific electrocardiographic signs, including conduction and rhythm disorder, repolarization disorders, and myocardial infarction. Thus, this study recommends monitoring by electrocardiography of patients with NHL, and especially the addition of transthoracic ultrasound before starting the treatment, given the cardiac toxicity of the drugs used [8].

In imaging, CT scan, magnetic resonance imaging (MRI), transthoracic ultrasound, transesophageal ultrasound, and sometimes positron emission tomography (PET) scan help to discover or guide the diagnosis. In current practice, imaging is requested first and helps in the etiological diagnosis by providing an overall view of the tumor involvement. However, its sensitivity is low, which explains the discrepancy between the clinical prevalence of cardiac metastases and the frequency of their discovery during autopsies [9]. In the literature, cardiac involvement of lymphomatous origin is described by transthoracic echography as fixed or mobile tumor vegetation, often multilobed, and more often located in the right cavities [10,11]. The transesophageal ultrasound allows a more detailed analysis that can guide the biopsy and the possibility of surgical resection [7]. However, MRI remains the most efficient examination because it allows the detection of lesions that have escaped ultrasound and CT scans and specifies the intracardiac and extracardiac limits with a sensitivity of more than 90% [7,11,12].

Histological confirmation remains difficult to obtain because most often patients are fragile, making biopsy/exeresis by thoracotomy challenging. Analysis of the pericardial puncture fluid provides histological evidence in 67% of cases [11]. However, its negativity does not eliminate the diagnosis of lymphoma. Thus sometimes in cases of disseminated disease with histological confirmation on other organs, the diagnosis of secondary cardiac involvements is strongly suspected on imaging without performing a diagnostic cardiac biopsy, as was the case for our patient. The most common histology is large B-cell lymphoma [13].

In general, the therapeutic management of cardiac involvements of various origins is discussed on a case-by-case basis depending on the patient's condition. When cardiac involvement is the only metastatic site, surgery can be considered mainly in the case of a significant risk of cardiac dysfunction or other complications [6,14,15]. However, it is often incomplete and the postoperative mortality rate is high (40%). In the case of disseminated disease, chemotherapy is proposed depending on the primary tumor [13]. In involvements of lymphomatous origin, treatment is mainly by chemotherapy, with or without radiotherapy, with poorer results compared to systemic disease without associated cardiac damage [7,16].

In general, the prognosis for cardiac metastases remains poor with survival of no more than four months according to the literature [5,17]. The prognosis for cardiac involvements of lymphomatous origin is also reserved, although the literature reports cases of prolonged remission [9,10,18].

## Conclusions

Cardiac involvements from NHL are rarely described in clinical practice because of their usually insidious progression. Moreover, their histological diagnosis is part of the dynamics of the diagnosis of systemic disease which is often disseminated at the time of their discovery, with a biopsy often not possible. Thus, cardiac ultrasound and MRI remain the best means of diagnosis. Management is essentially medical with a reserved prognosis despite the chemo-sensitivity of the lymphomatous disease itself.
